# The prevalence of BRCA1 mutations among young women with triple-negative breast cancer

**DOI:** 10.1186/1471-2407-9-86

**Published:** 2009-03-19

**Authors:** SR Young, Robert T Pilarski, Talia Donenberg, Charles Shapiro, Lyn S Hammond, Judith Miller, Karen A Brooks, Stephanie Cohen, Beverly Tenenholz, Damini DeSai, Inuk Zandvakili, Robert Royer, Song Li, Steven A Narod

**Affiliations:** 1 Department of Obstetrics and Gynecology, University of South Carolina School of Medicine, USA; 2 Department of Internal Medicine, Clinical Cancer Genetics Program, Ohio State University, Columbus, OH, USA; 3 Department of Obstetrics & GynecologyUniversity of Miami/Jackson Health System, Miami, Florida, USA; 4 Department of Internal Medicine, Division of Hematology and Oncology, Ohio State University, USA; 5 Division of Genetics, Medical University of South Carolina, Charleston, SC, USA; 6 Carle Clinic Medical Genetics, University of Illinois College of Medicine, Urbana, IL, USA; 7 Medical Genetics, St. Vincent Hospital, Indianapolis, IN, USA; 8 Geisinger Medical Center, Danville, PA, USA; 9 Joe Arrington Cancer Center, Lubbock, TX, USA; 10 Women's College Research Institute, Department of Public Health, The University of Toronto, Toronto, Canada

## Abstract

**Background:**

Molecular screening for BRCA1 and BRCA2 mutations is now an established component of risk evaluation and management of familial breast cancer. Features of hereditary breast cancer include an early age-of-onset and over-representation of the 'triple-negative' phenotype (negative for estrogen-receptor, progesterone-receptor and HER2). The decision to offer genetic testing to a breast cancer patient is usually based on her family history, but in the absence of a family history of cancer, some women may qualify for testing based on the age-of-onset and/or the pathologic features of the breast cancer.

**Methods:**

We studied 54 women who were diagnosed with high-grade, triple-negative invasive breast cancer at or before age 40. These women were selected for study because they had little or no family history of breast or ovarian cancer and they did not qualify for genetic testing using conventional family history criteria. BRCA1 screening was performed using a combination of fluorescent multiplexed-PCR analysis, BRCA1 exon-13 6 kb duplication screening, the protein truncation test (PTT) and fluorescent multiplexed denaturing gradient gel electrophoresis (DGGE). All coding exons of BRCA1 were screened. The two large exons of BRCA2 were also screened using PTT. All mutations were confirmed with direct sequencing.

**Results:**

Five deleterious BRCA1 mutations and one deleterious BRCA2 mutation were identified in the 54 patients with early-onset, triple-negative breast cancer (11%).

**Conclusion:**

Women with early-onset triple-negative breast cancer are candidates for genetic testing for BRCA1, even in the absence of a family history of breast or ovarian cancer.

## Background

The two major contributors to hereditary breast cancer are the cancer susceptibility genes BRCA1 and BRCA2 [1]. Genetic testing for BRCA1 and BRCA2 mutations has been established throughout North America and much of Europe. Not all women are candidates for testing – in general, testing is offered to a woman who has a probability of ten percent or greater for being positive for a mutation. An exception is made for women from particular ethnic backgrounds, such as Ashkenazi Jewish, French-Canadian or Polish women; in these groups, a small number of founder mutations is responsible for the majority of hereditary cancers [2-4]. For women in these groups, testing is inexpensive and eligibility criteria can be relaxed. However, in a mixed ethnic population, such as is found in most of the United States and Canada, testing involves the complete screening of the coding sequences of the two genes. In order to maximize both the efficiency of testing and the public health impact of a genetic screening program, many centers offer screening to women when the prior probability of finding a mutation is ten percent or greater. Several mathematical models such as BRCAPRO and BOADICEA can be used to estimate the prior probability of having a mutation [5,6]. These models consider age-of-onset and family history of cancer. Certain characteristics of the breast cancer can also be used to help predict the presence of a mutation. BRCA1-associated cancers are typically high-grade and are 'triple negative'; i.e. are negative for estrogen-receptor (ER), progesterone-receptor (PR) and HER2 expression [7,8]. The majority of triple-negative cancers exhibit the basal phenotype, i.e., they also express basal type cytokeratins (keratin 5 or keratin 6) or epidermal growth factor receptor (EGFR) (80% to 90% of triple-negative cancers are basal phenotype). The proportion of BRCA1-associated cancers that are of the 'basal phenotype' has been estimated to be 88% by Foulkes et al [7] and 57% by Lakhani et al. [8]. Furthermore, the proportion of BRCA1-associated cancers that are ER-negative (one of the component features) diminishes with increasing age-of-onset. Foulkes et al found that 81% of BRCA1-associated breast cancers diagnosed before age 45 were ER-negative, compared to 62% of cancers in women diagnosed after age 65 [9]. In contrast to the high proportion among BRCA1-associated breast cancer, only about 15% of all women with breast cancer have triple-negative cancers [10]. Therefore, this subgroup of women is expected to be enriched for BRCA1-mutation carriers. In this study, we estimate the proportion of BRCA1 mutation carriers among women diagnosed at age 40 or younger with triple-negative breast cancer, without a significant family history of cancer.

## Methods

Cases for study were identified from two hospital systems (the Palmetto Health Richland/Baptist Hospitals, Columbia SC and the Cancer Genetics Clinic of Ohio State University, Columbus) and through genetic counselors affiliated with the National Society of Genetic Counselors Cancer Special Interest Group. Women diagnosed with breast cancer at age 40 years and younger and who did not have a significant family history of breast or ovarian cancer were chosen for study. We also excluded patients of Ashkenazi Jewish heritage because they would be eligible for routine genetic testing (founder mutations) in any cancer center and because we do not expect to find non-founder mutations in this population. A total of 97 patients were submitted for evaluation, of which 58 were eligible for the study. Women were excluded if there was insufficient documentation of triple-negative status to include them in the study (n = 32) or if they had a positive family history of cancer (n = 6) or if the age of diagnosis was missing (n = 1). Twenty-eight patients were submitted from the Palmetto Health Richland and Baptist Hospitals. Women were identified though the Palmetto Health Cancer Directory or at presentation to a weekly breast cancer conference. A woman was eligible if her medical records indicated that her breast carcinoma was grade III and was negative for ER, PR and HER2. HER2 overexpression was defined as moderate to strong staining that totally encircles the cell membrane (2+ or 3+). Women with a cancer diagnosis within three years of study initiation were invited to participate. These 14 women were mostly from South Carolina. Nineteen patients were submitted from Ohio State University: these women were identified from a database of 1300 breast cancer patients who had been accrued to a separate research protocol. They were diagnosed with breast cancer at one of three hospitals in the Columbus, OH area, between May 2003 and August 2006. Informative cases were chosen for being high-grade, triple-negative breast cancers diagnosed at age 40 and younger. Twenty-five patients were recruited through the National Society of Genetic Counselors Cancer Special Interest Group. An email notification was posted on the society list-serve (NSGC Cancer-SIG). This list-serve is read by approximately 300 genetic counselors who specialize in cancer. The notice solicited breast cancer patients who diagnosed at age 40 or younger, had no cancer family history and who were 'triple-negative'. Twenty-two counselors submitted patients. All patients accrued through the NSGC Cancer-SIG were interviewed by a genetic counselor and a three-generation pedigree was drawn. The study was approved by the institutional review board (IRB) of the Palmetto Health Richland/Baptist Hospitals IRB (Columbia, SC 29201) and of the Women's College Research Institute.

### Laboratory methods

DNA was extracted from whole blood lymphocytes using standard methodology. We evaluated the entire coding sequence of BRCA1 and the large exons 10 and 11 of BRCA2 for mutations. DNA was screened for two common BRCA1 alterations (185delAG and 5382insC) and one BRCA2 alteration (6174delT) by rapid fluorescent multiplexed-PCR analysis, FMPA [11]. All patients were screened for the BRCA1 exon-13 6 kb duplication [12]. BRCA1 exon 11, and BRCA2 exons 10 and 11 were screened using protein truncation test, PTT (TNT™ rabbit reticulocyte lysate system, Promega, and [35S]methionine/cysteine, New England Nuclear). All other BRCA1 exons, with the exception of exons 1a/b and 4, were also scanned by fluorescent multiplexed denaturing gradient gel electrophoresis, DGGE [13]. The first 2 kb at the beginning and the end of exon 11 were also included in the DGGE analysis, as were all exon-intron boundaries. All variants identified by either PTT or DGGE were confirmed by direct sequencing. These methods are expected to identify almost all BRCA1 mutations and 70% of BRCA2 mutations which are identifiable through direct sequencing.

## Results

A total of 58 women with triple-negative cancer was eligible for testing, but four samples were of poor quality and these patients were excluded, leaving 54 patients. The mean age of cancer diagnosis was 34.7 years (range 24 to 40 years). None of the patients had a significant family history as defined by the American Society of Clinical Oncology [14]. No patient had a past history of ovarian cancer and no patient had a first-degree relative with ovarian cancer or breast cancer. No patient had a family history of male breast cancer. Two patients had bilateral breast cancer; in both instances the triple-negative tumour was the first tumour to be diagnosed and neither had a family history of breast or ovarian cancer.

Six deleterious mutations were identified in the 54 patients (11%); five in BRCA1 and one in BRCA2 (table 1). The 5382insC mutation was seen on two occasions and the four other mutations were seen one time each. All mutations were protein-truncating. A BRCA1 mutation was identified in 5 of 34 white patients (15%) and none of 20 patients of other ethnicities (ten African-American, six Hispanic, two Asian, two mixed). The single BRCA2 mutation was found in an African-American woman (4936delAG). A mutation was found in 0 of 11 women diagnosed with breast cancer at or before age 30 and in 6 of 43 women (14%) diagnosed between ages 31 and 40. Nine additional BRCA1 variants of no, or unknown, clinical significance were also identified (table 2).

**Table 1:** Deleterious mutations identified among fifty-four triple-negative cases:

Gene	Exon	Mutation	Mutation Type	Age of Onset	Ethnic Group
BRCA1	11	1294del40	Deletion	35	Caucasian
BRCA1	11	2800delA	Deletion	32	Irish/Scottish
BRCA1	15	4731C>T	Nonsense	36	Caucasian
BRCA1	20	5382insC	Insertion	38	Caucasian
BRCA1	20	5382insC	Insertion	39	Caucasian
BRCA2	11	4936delAG	Deletion	39	African American

**Table 2:** Unclassified variants identified among fifty-four triple-negative cases:

Gene	Exon	Mutation	Designation	Mutation Type	Age of Onset	Ethnic Group
BRCA1	22	676C>A	S186Y	Missense	36	Puerto Rican
BRCA1	16	4956A>G	S1613G	Missense	40	African American
BRCA1	15	4654G>T	S1512I	Missense	36	Caucasian
BRCA1	11	1186A>G	Q356R	Missense	40	Iranian

## Discussion

These data support the position that early-onset triple-negative breast cancer is an indicator that can be used to help to identify candidates for BRCA1 mutation testing. Our study is relatively small (54 patients) but we confirm similar observations made by others. To date, no single study has been large or definitive, and therefore it is important to consider the results of all studies in aggregate. Lidereau et al found that 6 of 70 women (9%) with breast cancer diagnosed at age 35 or below, unselected for family history, carried a BRCA1 mutation [15]. However, the proportion with mutations was 29% (4 of 14) for those with ER-negative, high-grade tumours, compared to only 4% (2 of 56) among women with other tumour types (odds ratio = 11; p = 0.007). Among women in our study who were diagnosed before the age of 35, the mutation prevalence was 8.3%. In a study of 254 white women from the UK, diagnosed with breast cancer before age 36, a germline BRCA1 or BRCA2 mutation was identified in only 6% (ref 16). Of the 15 women with germline mutations, only one had a family history of breast or ovarian cancer. In this early study, the study sample was not subdivided by ER-status or other factors.

A number of studies suggest that breast cancers associated with BRCA1 mutations are likely to be triple-negative and the majority of these are also the basal phenotype [7,8,17-19]. Basal-like tumours express certain cytokeratins characteristic of the 'basal' layer myoepithelial cells lining the terminal duct lobular unit (namely cytokeratins 5, 6, 14 and 17) [20]. Basal-like tumours are usually high-grade, exhibit comedeonecrosis, pushing borders and an inflammatory lymphocytic infiltrate.

Other studies included women who were selected for family history or ethnic group. Chang *et al. *studied women with familial breast cancer (at least one first-degree relative with either breast or ovarian cancer) diagnosed before age 45 [17]. They found that 6 of 24 (25%) patients with an ER-negative, high-grade tumour had a germline BRCA1 mutation. Haffty *et al. *that reported that eight of 34 women with triple-negative breast cancer had a BRCA1 mutation (24%), but these included women with a strong family history of breast cancer [18]. Foulkes *et al. *found that of 24% of 72 Ashkenazi Jewish patients diagnosed before age 65 with high grade, ER-negative, HER2-negative breast cancers had a germline BRCA1 mutation [7]. However, the frequency of mutations is known to be high in this group. Given that all Ashkenazi Jewish women with breast cancer are candidates for BRCA mutation testing, we excluded this group from the present study.

Our study has several limitations. Our subjects were derived from various sources, including a population-based series of cases (Ohio), and from a US hospital (North Carolina) and from women who presented for genetic risk assessment to various clinics across the country. The determination of grade, ER, PR and HER2 status was based on review of pathology report and there was no central pathology review (however, this situation accurately reflects actual clinical practise). The purpose of this study was to estimate the prevalence of BRCA1 mutations and the entire BRCA2 gene was not screened. However, the triple-negative phenotype is not characteristic of BRCA2-associated breast cancers. We expect that the majority of BRCA mutations diagnosed in the triple-negative, family history negative subgroup cancers will be BRCA1. In most situations, genetic counsellors will offer to test for both genes, and if a woman is insured, testing for both genes will be covered. However, for women who do not have full coverage, or in countries where resources are limited, some may wish to test triple-negative patients for BRCA1 only.

All five BRCA1 mutations were seen in white women between the ages of 31 and 40; in this subgroup the mutation prevalence was 17%. It is interesting that no mutation was identified in a woman diagnosed before age 30; however this subgroup was small and it is in not clear if this is a chance finding. Further studies will be useful in determining if there are other genetic markers of very early-onset breast cancer.

The criteria used to offer genetic testing to a patient varies from center to center, but in general, testing is usually offered to a woman if the probability of finding a mutation exceeds ten percent [14]. Most guidelines for selecting patients rely on family history, but in many centers, genetic testing is routinely offered to all women diagnosed with breast cancer under the age of 40. In the absence of a family history, fewer than ten percent of patients in this group are expected to carry a BRCA mutation.

## Conclusion

Our study indicates that young women with a high-grade triple-negative cancer and no family history of cancer may be candidates for genetic testing. However, women with other histologic forms of cancer (e.g. ER-positive or HER2-positive) and with no family history are unlikely to carry a mutation.

## Authors' contributions

SRY conceived of the study and helped draft manuscript

RTP, TD, CS, LSH, JM, KAB, SC, BT, DD, coordinated the activities at their centers

IZ, RR, SL carried out molecular assays

SAN oversaw the data collection and statistical analysis and helped draft the manuscript.

## Pre-publication history

The pre-publication history for this paper can be accessed here:

http://www.biomedcentral.com/1471-2407/9/86/prepub
